# Two approaches to the use of benzo[c][1,2]oxaboroles as active fragments for synthetic transformation of clarithromycin^*^

**DOI:** 10.1080/14756366.2016.1261129

**Published:** 2017-01-18

**Authors:** Gennady B. Lapa, Elena P. Mirchink, Elena B Isakova, Maria N Preobrazhenskaya

**Affiliations:** a Blokhin Cancer Center, Moscow, Russia;; b Pirogov Russian National Research Medical University (RNRMU), Moscow, Russia;; c Gause Institute of New Antibiotics, Moscow, Russian Federation

**Keywords:** Antibiotic, macrolactone, macrolide, clarithromycin, benzoxaborole, 1-hydroxy-1,3-dihydrobenzo[c][1,2]oxaborole, antibacterial, conjugates of antibiotics

## Abstract

Clarithromycin (active against Gram positive infections) and 1-hydroxy-1,3-dihydrobenzo[c][1,2]oxaborole derivatives (effective for Gram negative microbes) are the ligands of bacterial RNA. The antimicrobial activities of these benzoxaboroles linked with clarithromycin at 9 or 4″ position were compared. Two synthetic pathways for these conjugates were elaborated. First pathway explored the substitution of the C-9 carbonyl group of macrolactone’s cycle via oxime linker, the second direction used the modification of the 4″-O-group of cladinose via the formation of carbamates of benzoxaboroles. 4″-O-(3-S-(1-Hydroxy-1,3-dihydro-benzo[c][1,2]oxaborole)-methyl-carbamoyl-clarithromycin showed twofold decrease in MICs for *S. epidermidis* and *S. pneumoniae* than clarithromycin. 4″-O-Modified clarithromycin demonstrated an efficacy against Gram positive strains only. Compounds with C-9 substitution were more active than 4″-O-substituted antibiotics for susceptible strains *E. coli* tolC and did not exceed the activity of initial antibiotics.

## Introduction

The role of new semisynthetic antibiotics increases with the emergence and spread of resistant strains of pathogens, since the process of introduction of totally new antibiotics is durable and does not guarantee success in clinical research^1^. We believe that research of new ways of transformation of wide used antibiotics could significantly speed up overcoming of insusceptible strains accumulation^2^. Recent advances in chemistry and antibacterial chemotherapy of 1-hydroxy-1,3-dihydrobenzo[c][1,2]oxaborole heterocycles (BB) reveal a new group of antibiotics with new mechanism of action^3^. Some of these benzoxaboroles inhibit cytoplasmic leucyl-tRNA synthetase by the formation of a stable tRNA(Leu)-benzoxaborol adduct in the editing site of the enzyme of predominantly Gram negative strains (Gr-)^4^. The ribosomal RNA is the main target in the mechanism of action of clarithromycin (CLA) – erythrolide antibiotics^5^^,^^6^. Thus, both types of active compounds disrupt the biosynthesis of bacterial proteins as the ligands of RNA. The P orbital reactivity of boron of BB template provides a variety of modalities of binding to nucleophiles. This BB template can form a reversible tetrahedral adduct with 2′-3′-cis-hydroxyls^3^^,^^4^^,^^7^, 2′-hydroxyl^8^ of ribonucleotides and even hydroxyl of serine in active center of enzymes^9^. From this point of view, BB template could be a good moiety to enhance the structural diversity of CLA as a ligand of bacterial rRNA. Benzoxaboroles would increase the number of binding sites to the target RNA and could be attractive fragments among other ligands of RNA for such conjugates. The achievements in the synthesis of new erythrolides have shown a little progress in this direction^10^^,^^11^. One should take into account the lipophilicity of final semi-synthetic erythrolides since these antibiotics penetrate the bacterial wall passively^10–12^. Additionally, both antibiotics are active at about the same MIC values^4^^,^^7^^,^^12^. BB are active against Gr − strains but CLA acts on Gr + microbes which may lead to increase antibacterial spectra of these semisynthetic antibiotics on the base of CLA^11^^,^^12^. A few successful strategies of chemical transformation of erythrolides are based on the increase of the permeability through the bacterial wall and resistance in acidic media, as well as the protection of the sites of the molecule exposed to enzymatic inactivation^13^^,^^14^. In this study, we describe the synthesis of new clarithromycin–benzoxaboroles conjugates via two approaches, as well as their anti-bacterial properties and the structure–activity relationship (SAR).

## Materials and methods

All chemicals were from Sigma-Aldrich or Acros (USA) and were used without further purification. Compounds BB-A and BB-B were provided by prof. Preobrazhenskaya from previous collaborations^2^ and ee of BB-A was accepted as provided. Analytical TLC for checking the homogeneity of the compounds was made using TLC on silica gel-protected aluminum sheets (Type 60 F254, Merck) with chloroform-methanol as a mobile phase and the spots were detected by exposure to a UV-lamp at 254 nm. The structures of all synthesized compounds were confirmed by 400 MHz ^13^C-NMR spectra (Varian VXR-400) and high-resolution ESI mass-spectrometry (microTOF-Q II, Bruker Daltonics GmbH). ^13^C-NMR spectra were recorded in DMSO-d_6_ or in CDCl3. Purity was checked by HPLC (column Kromasil C-18, 250 × 4.5 mm, PDA, mobile phase 0.03% HCOOH (pH = 3) or 0.03% NH4COOH pH = 7.8, gradient with acetonitrile: from 10 to 95 w/w%. Final products were purified chromatographically. On the first stage they were purified by column chromatography on silica gel Merck 60 (using ISCO instrument with detector at 254 nM). About 10 or 20 mL of sorbent was used for 100–250 mg of reaction mixture. The gradient CHCl3–MeOH–NH4OH (0.1%) was used for elution to give compounds of 60–80% purity. For further purification, pTLC method on Merck 40F 254 plates was used with mobile phase from CHCl3–MeOH–NH4OH (0.1%).

### Computer models

**Figure 1.**
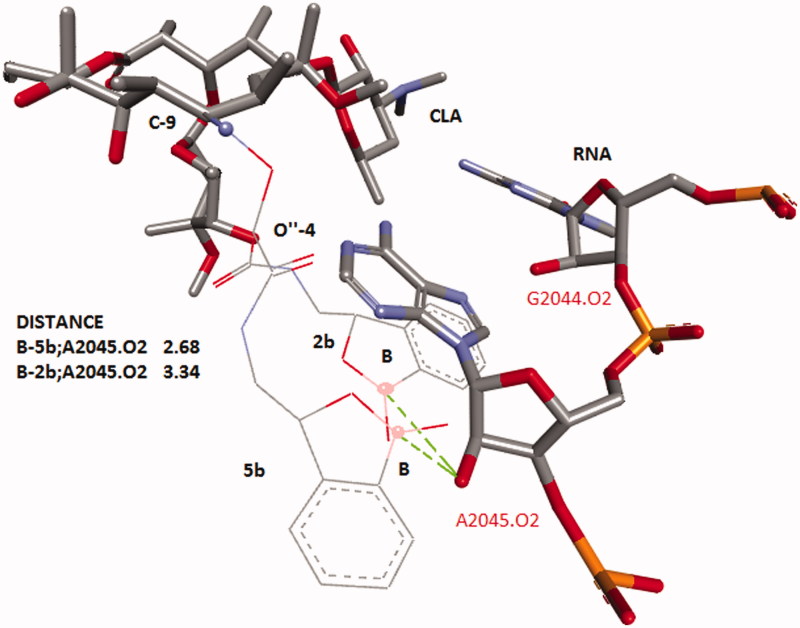
Alignment of conformers of CLA with the benzoxoboroles substituent at C-9 and 4″-O. Structure of CLA’s core was taken from 1J5A.pdb. Benzoxoborole tails were optimized in RNA’s environmental and the structure of RNA was omitted. Distances (in angstroms) are shown as dashed lines.

Computer models were created in DS ViewerPro 6.0 (Accelrys, San Diego, CA). The template of clarithromycin was taken from 1J5A.pdb and the structures of appropriate benzoxaboroles with the linkers were added to this template. Final model was cleaned by the option of “clean structure” which used a fast, Dreiding-like force field to quickly optimize the geometry of all or selected structures in the 3D Window. The elements, bond orders, number of bonds, and valences are taken into consideration when the terms of the energy equation are calculated. The molecules of antibiotic and the nearest parts of RNA were selected then the molecules are cleaned with the van der Waals effects of the other selected molecules included in the calculation. An alignment of two of conformers with different position of BB-tails was made along macrolacton’s core (Figure 1).

### Chemistry

#### Method for 9-(O-carboxymethyl-oxime)-clarithromycin (1)

This mixture carboxymethoxylamine hemihydrochloride (2.5 mmol) and clarithromycin (1.0 mmol) in methanol (7.0 ml) was stirred at 30–35 °C for 2 days. Reaction mixture was diluted with brine and extracted with chloroform 15 ml for 3 time, combined extract was washed with brine, dried over anhydrous Na_2_SO_4_ and evaporated *in vacuo* to afford a crude product. The crude product was purified by flash-chromatograph on silica gel with chloroform-methanol to afford a pure product as a mixture syn- anti-isomers which was used for next step.

#### 9-syn/anti-(O-carboxymethyl-oxime)-clarithromycin (**1**)

Уield 60% after flash-chromatography. ^13^C-NMR (DMSO-d_6_): 8.9, 10.6, 11.8, 15.1, 15.7, 16.7, 17.1, 18.5, 18.7, 19.8, 20.8, 21.3, 26.0, 30.3, 34.7, 38.5, 39.8, 44.4, 44.7, 48.6, 48.9, 50.4, 56.0, 64.5, 64.7, 65.0, 65.0, 66.6, 66.7, 69.2, 69.5, 70.1, 71.2, 72.5, 73.6, 76.2, 76.7, 77.1, 77.2, 77.3, 78.1, 78.4, 79.3, 95.6, 95.7, 101.7, 101.9, 169.4, 171.9, 172.8, 175.2, 175.4. HPLC purity 93.23% for two main peaks at 230 nm, (1:1 ratio of E- and Z-isomers). MW Calc. for C_40_H_72_N_2_O_15_ 820.4933. Found in ESI-ms 819.4840 (M − H) 100% or 821.5073 (M + H) 100%.

#### General method for amides of 9-(O-carboxymethyl-oxime)-clarithromycin (2)

The mixture of acid 1 (1 mmol), amine (A or B) (1.5 mmol), PyBOP (1.5 mmol), triethylamine (5 mmol) in dichloromethane 15 ml was stirred at room temperature for 24 h. Reaction mixture was diluted with water to 15 ml, pH of aqueous layer was adjusted to 5.5–6.8 by dry citric acid. This mixture was extracted with chloroform for 3 times, combined extract was dried over anhydrous Na_2_SO_4_, filtered and concentrated *in vacuo* to afford a crude product. The crude product was purified by chromatography on silica gel.

#### [7-(1-Hydroxy-1,3-dihydro-benzo[c][1,2]oxaborole)methyl]amide of (E/Z)-9-clarithromycin methoxyoxime (2a)

Yield 32% after pTLC. ^13^C-NMR (DMSO-d_6_): 9.0, 10.5, 10.6, 12.1, 14.9, 15.2, 15.7, 16.8, 17.3, 18.4, 18.7, 18.8, 20.8, 21.4, 26.1, 30.0, 34.7, 38.4, 40.2, 40.6, 44.4, 48.8, 50.3, 64.5, 64.9, 65.0, 65.6, 69.3, 69.8, 70.5, 71.7, 72.5, 73.6, 74.5, 77.0, 77.2, 78.2, 78.4, 78.9, 95.6, 95.8, 101.9, 102.2, 154.1, 168.7, 169.1, 171.0, 175.1, 175.5. HPLC purity 98.17% for two main peaks at 230 nm (1:1 ratio of E- and Z-isomers), system B, RT 15.2 and 15.8 min. MW Calc. for C_48_H_80_BN_3_O_16_ 965.5631. Found in ESI-MS 966.5719 (M + H)^+^.

#### [(3-S-(1-Hydroxy-1,3-dihydro-benzo[c][1,2]oxaborole)methylamide of (E/Z)-9-сlarithromycin methoxime (**2b**)

Yield 9% after 4-th pTLC. ^13^C-NMR (DMSO-d_6_): 8.9, 10.4, 10.6, 14.5, 15.1, 15.7, 16.7, 18.6, 18.7, 19.5, 20.6, 20.8, 20.9, 21.2, 21.3, 27.1, 30.0, 34.6, 36.0, 40.0, 40.41, 45.25, 48.8, 49.2, 59.3, 64.4, 64.8, 66.7, 71.7, 72.5, 72.5, 76.9, 77.0, 77.1, 78.0, 79.0, 79.9, 80.7, 95.3, 95.7, 101.6, 101.9, 120.1, 125.2, 127.1, 134.8, 136.8, 144.9, 163.5, 168.7, 173.3, 176.4, 178.7. HPLC purity 84.9% for two main peaks at 274 nm (1:1 ratio of E- and Z-isomers), system A, Rt 12.6 and 13.0 min. MW Calc. for C_48_H_80_BN_3_O_16_ 965.5631. Found in ESI-ms 966.5776 (M + H)^+^.

#### 2′-O-Acetyl-4″-O-acylimidazolylclarithromycin (**4**)

Acetic anhydride (4.0 mmol, 2.0 equiv) was added dropwise to a mixture of clarithromycin (1.5 g, 2.0 mmol) and NaHCO_3_ (0.85 g, 10.0 mmol) in dichloromethane (10 mL) at 5–10 °C. The resulting solution was allowed to stir for 24 h at the room temperature. The reaction was quenched with 5% aqueous NaHCO_3_ (20 mL) and the aqueous layer was extracted with dichloromethane (2 × 10 mL). The combined organic layers were dried over anhydrous Na_2_SO_4_, filtered through 1 ml silica gel and concentrated *in vacuo* to afford pure product^3^ as white small crystals. Et_3_N (4.5 mmol) and 1,1′-carbonyldiimidazole (5.0 mmol) were added to a solution of the acyl intermediate (2.0 mmol) in toluene (25 mL). The resulting solution was stirred at room temperature for 48 h. The reaction was quenched with saturated NaHCO_3_ (20 mL) and the aqueous layer was extracted with toluene (3 × 10 mL). The combined organic layers were dried over anhydrous Na_2_SO_4_, filtered through 1 ml silica gel, and concentrated *in vacuo* to afford 95.0% of **4** as white foam.

#### 2′-O-Acetyl-clarithromycin (**3**)

Yield 91%. ^13^C-NMR – APT: 8.9 (n, 4-CH3), 10.5 (n,14-CH3), 12.2(n,10-CH3), 15.9 (n, 2-CH3), 16.0 (n, 12-CH3), 17.6 (n, 8-CH3), 18.6 (n, 5″-CH3), 19.8 (n, 6-CH3), 20.9 (p, C-14), 21.2 (n, 3″-CH3), 21.4 (n, 5′-CH3), 21.5 (n, CH2), 30.4 (p, C-4′), 34.8 (p, C-2″), 37.1 (n, C-10), 38.7 (n, C-4), 38.6 (p, C-7), 40.5 (n, CH3–N–CH3), 44.9 (n, C-2), 45.1 (n, C-8), 49.3 (n, 3″-OCH3), 50.4 (n, 6-OCH3), 63.4 (n, C-3′), 65.9 (n, C-5″), 67.9 (n, C-5′), 69.0 (n, C-11), 71.3 (n, C-2′), 72.7 (p, C-3″), 74.1 (p, C-12), 76.5 (n, C-13), 77.7 (n, C-4″), 78.0 (n, C-3), 78.2 (p, C-6), 80.4 (n, C-5), 95.7 (n, C-1″), 100.2 (n, C-1′), 170.0 (p,), 175.5 (p, C-1), 221.0 (p, C-9). MW Calcd for C_40_H_71_NO_14_ 789.4875. Found in ESI-ms 790.4898 (M + H)^+^.

#### General methods for 4″-O-arylalkylcarbamoyl clarithromycin derivatives (**5a,b**)

DBU (0.33 mL, 2.25 mmol) and corresponding arylalkylamine (4.5 mmol) were added to a solution of **4** (1.5 g, 1.70 mmol) in DMF (15 mL). The resulting solution was stirred for 7 h at 60 °C. The reaction was quenched with water (15 mL), pH of aqueous layer was adjusted to 5.5–6.8 by dry citric acid and extracted with ethyl acetate (2 × 15 mL). The combined organic layers were washed with brine, dried over anhydrous Na_2_SO_4_, filtered and concentrated *in vacuo* to afford a crude product. A solution of the above crude product in methanol (15 mL) was heated to 45 °C and stirred for 12 h at the same temperature. After concentrating the reaction solution *in vacuo*, the residue was purified by flash chromatography (chloroform/methanol, 25:1) to give compounds **5a, b**.

#### 4”-O-(7–(1-Hydroxy-1,3-dihydro-benzo[c][1,2]oxaborole)methyl)carbamoyl-clarithromycin (**5a**)

Yield 35% after pTLC. ^13^C-NMR: 8.9, 10.4, 11.8, 15.1, 15.6, 17.0, 17.7, 18.3, 19.9, 20.2, 20.7, 21.3, 30.1, 34.7, 38.1, 38.4, 40.3, 42.5, 43.5, 44.3, 48.9, 50.2, 63.0, 64.5, 66.35, 67.19, 68.9, 69.8, 70.5, 72.3, 74.1, 76.0, 77.4, 77.9, 78.0, 79.0, 95.7, 101.9, 119.6, 123.7, 128.0, 130.7, 143.6, 154.0, 156.5, 175.1, 218.6. HPLC purity 94.46% at 225 nm. MW Calc. for C_47_H_77_BN_2_O_16_ 936.5366. Found in ESI-MS 937.5481 (M + H)^+^.

#### 4”-O-(3-S-(1-Hydroxy-1,3-dihydro-benzo[c][1,2]oxaborole)methyl)carbamoyl-clarithromycin (**5b**)

Yield 18% after second pTLC. ^13^C-NMR: 8.9, 10.4, 11.8, 15.1, 15.6, 17.0, 17.7, 18.3, 19.9, 20.2, 20.7, 21.3, 30.1, 34.7, 38.1, 38.4, 40.3, 42.5, 43.5, 44.3, 48.9, 50.2, 63.0, 64.5, 66.3, 67.1, 68.9, 69.8, 70.5, 72.3, 74.1, 76.0, 77.4, 77.9, 78.0, 79.0, 95.7, 101.9, 119.6, 123.73, 128.09, 130.7, 143.6, 154.0, 156.5, 175.1, 218.6. HPLC purity 91.48% at 268 nm. MW Calc. for C_47_H_77_BN_2_O_16_ 936.5366. Found in ESI-MS 937.5422 (M + H)^+^.

### Biology

Minimum inhibitory concentration (MIC) of the synthesized compounds **1**–**5** was evaluated *in vitro* against panel of microbes by well-documented bioassays in μg/ml^2^^,^^13^^,^^14^. Briefly, samples were diluted in the solution of DMSO–0.9% NaCl (15:85 by volume). MIC was determined by broth micro-dilution method using the Mueller–Hinton broth, as recommended by NCCLS procedures. The MIC’s were determined to be the lowest concentration of compounds that inhibited microbial growth by more than 50% of the positive growth control. Broth microdilution assays were performed in triplicate for each strain. The whole assay was repeated twice (after two weeks) to be sure in stability of dry **2a**,**b** and **5a**,**b** at 4 °C. Results were usually identical. Standard error of MIC was less than 0.1.

## Results and discussion

**Scheme 1.**
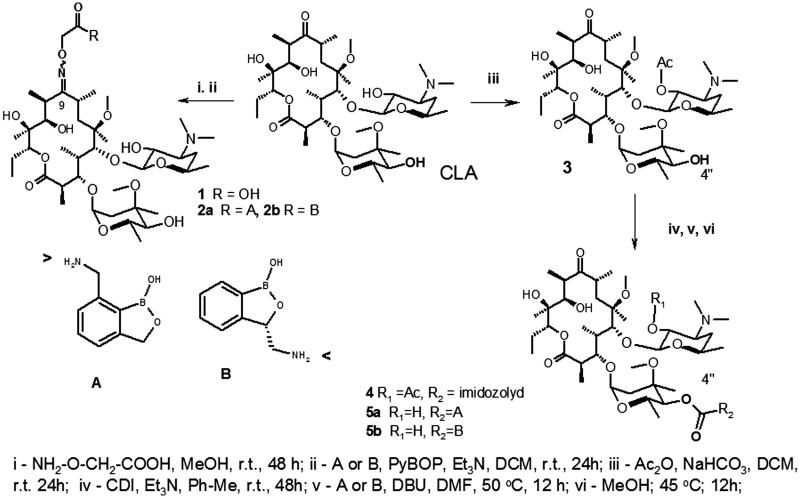
Synthesis of clarithromycin derivatives **1**–**5**.

The combinations of known antibiotics and active fragments of small molecules targeted to the same receptor into pathogenic microbes is one of the ways to create new molecules and overcome resistance^1^^,^^2^^,^^12^. Since benzoxaboroles (BB) can bind to the 2′-oxygen atoms of RNA, then BB could be the appropriate fragments for synthetic transformation of CLA. Both molecules are the ligands of bacterial RNA^3–8^, both molecules have similar range of MIC’s. However, BB and CLA act on different types of bacterial RNA and have different antimicrobial spectra. CLA is active against Gr + strains, but BB have shown efficacy against Gr- pathogens. Could the combination of CLA and BB increase antibacterial spectrum? Could BB fragments linked with CLA reach to the 2′-oxygen atoms of RNA in our models? We have used data of PDB file 1J5A and several considerations on the base of molecular models to answer these questions (Figure 1). On the base of crystallographic CLA’s conformation, analysis of possible chemical transformation of CLA and available compounds BB-A and BB-B with short linkers (Scheme 1) we made two models. First part of this model was based on the substitution of the carbonyl group at the C-9 position of macrolactone cycle (Figure 1). The second part of this model showed several possibilities in the case of the addition of BB fragments to the 4″-O-moiety of cladinose in CLA (Figure 1). Both models demonstrated that the linked benzoxaboroles were located adjacent to 2′hydroxyl of the RNA sugar-phosphate backbone. This simple model did not provide a rationale for the selection of one linkage or BB orientation over another. Therefore, we used two synthetic approaches for the synthesis of these conjugates to resolve this raised question.

The first approach is based on the preparation of 9-carboxymethoxyoxime of clarithromycin **1** and formation of amide of **1** by the reaction of **1** and primary aliphatic amino group of BB (Scheme 1). This pathway could provide two general aims: to make more acid stable molecule of macrolactone antibiotic and introduce antibacterial BB-fragment. We demonstrated in the first part of the model (Figure 1) that E- or Z-configurations of CLA-oximes **1** did not provide any difference in the possibility of BB-fragment to reach RNA’s sugar-phosphate chain. The synthesized oxime **1** represents a mixture of E- and Z-isomers. It has two main peaks in HPLC and the results of ESI-MS showed one peak with an appropriate molecular weight. ^13^C NMR spectra demonstrated the set of signals of E- and Z-isomers. In ^13^C NMR spectra of amides **2a,b** there were two sets of signals of carbons of amide groups and low field signals of aromatic atoms. Both antibiotics **2a,b** were used as the mixtures of E/Z-isomers for biological evaluations.

The second synthetic approach is based on the known synthetic procedure which included four steps: the protection of 2′-hydroxyl of desosamine by the acylation **3**, formation of 4″-O-imidazole carboxylate as an active intermediate **4**, formation of carbamates and final deprotection of 2′-hydroxyl group^13^^,^^14^. The formation of carbamates **5** was accomplished via the reaction of 4″-O-(imidazole-1-yl)carboxylate-2′-O-acetyl-clarithromycin **4** with a primary aliphatic amino group of BB. The structures of known intermediates **3** and **4** were supported by NMR and ESI-MS. Final **5a,b** carbamates were purified by two-step procedures with preliminary flash chromatography on silica gel and final preparative TLC to give compounds for analytical analysis and the evaluations of MIC’s (Tables 1 and 2).

**Table 1.** MIC’s (μg/mL) of the compounds against test-strains of Gr + and Gr − microbes^a^.

Compound ID	*S. aureus* 25923	*S. epidermidis* 12228	*S. pneumoniae* 49619	*E. feacalis* 29212	*E. coli* 25922	*K. pneumoniae* 13883	*S. cholerasuis* 14028	*P. aeruginosa* 27853
**2a**	1.0	1.0	8.0	8.0	>64.0	>64.0	>64.0	>64.0
**2b**	2.0	0.5	4.0	8.0	>64.0	>64.0	>64.0	>64.0
**5a**	2.0	2.0	2.0	4.0	>64.0	>64.0	>64.0	>64.0
**5b**	2.0	0.5	0.5	4.0	>64.0	>64.0	>64.0	>64.0
**CLA**	1.0	1.0	4.0	4.0	>64.0	>64.0	>64.0	>64.0
**BB A**	>64.0	>64.0	>64.0	>64.0	16.0	16.0	16.0	16.0
**BB B**	>64.0	>64.0	>64.0	>64.0	1.0	1.0	1.0	1.0

a Standard error of MIC was less than 0.1.

**Table 2.** MICs (mg/mL) *E. coli* K12 WT and tolC strains of *E. coli*^a^.

Compound ID	WT	tolC	tolC pUC57 msr-E, mphE	tolC pUC erm42
**2b**	>64	8	>64	16
**5a**	>64	8	>64	16
**5b**	>64	16	>64	32
**AN9246**	32	1	>64	2
**Erythromycin**	>64	2	>64	8
**Telithromycin**	16	0,5	>64	0,5

a Standard error of MIC was less than 0.1.

The antimicrobial evaluation demonstrated that oxime **1** did not possess any significant activity. In general, all compounds had the MIC’s in about the same range that initial CLA, only **5b** has revealed activity significant better than CLA for *S. pneumoniae*. The increased efficacy of BB-CLA antibiotics in *in vitro* tests for *Staphylococcus* and *Streptococcus* species was shown for **5a,b**. We can conclude that compounds **5a,b** are more active against Gr + strains than the compounds substituted at C-9 **2a,b**. Compound **5b** is more active against Gr + strains than **5a** and **2b** is better than **2a** in our *in vitro* tests. The activity of CLA-BB antibiotics against susceptible Gr- strains of *E. Coli* tolC and tolC pUC erm42 has shown completely opposite profile. Antibiotics **2b** were more active than **5b** in this test. Perhaps, this trend reflected an action of our compounds on fine distinction in the protein biosynthesis inhibition of Gr − and Gr + microbes, since, the structures of ribosomes of Gr − and Gr + microbes have tiny differences^10^. Probably, new compounds CLA-BB were acted in the same mode as a natural antibiotic, since new conjugates did not show sharp differences of activity from initial CLA.

## Conclusion

We developed two synthetic pathways for the preparation of various benzoxaborole–clarithromycin conjugates via functionalization of the C-9 and 4″-O positions of clarithromycin. These semisynthetic antibiotics were active only against Gr + strains and showed no broad action simultaneously on Gr − and Gr + strains. The addition of BB fragment to the 4″-O-position of cladinose gave more active compounds against Gr + strains than the substitution of the C-9 carbonyl group. In the opposite manner, the substitution of the C-9 position of CLA was preferable for activity against susceptible Gr− *E. Coli* TolC and tolC pUC erm42 strains than the addition to 4″-O-moiety.
